# Supplementary material for: Accessible and accurate cytometry analysis of adherent cells using fluorescence microscopes
Source: Sci Rep. 2025 May 28;15:18691. doi: 10.1038/s41598-025-01957-5 (PMC12120079; doi:10.1038/s41598-025-01957-5)
Supplement: Supplementary file 1 — Supplementary Information. [file 41598_2025_1957_MOESM1_ESM.pdf]

Accessible and accurate cytometry analysis of adherent cells using fluorescence microscopes: SUPPLEMENTARY MATERIALS

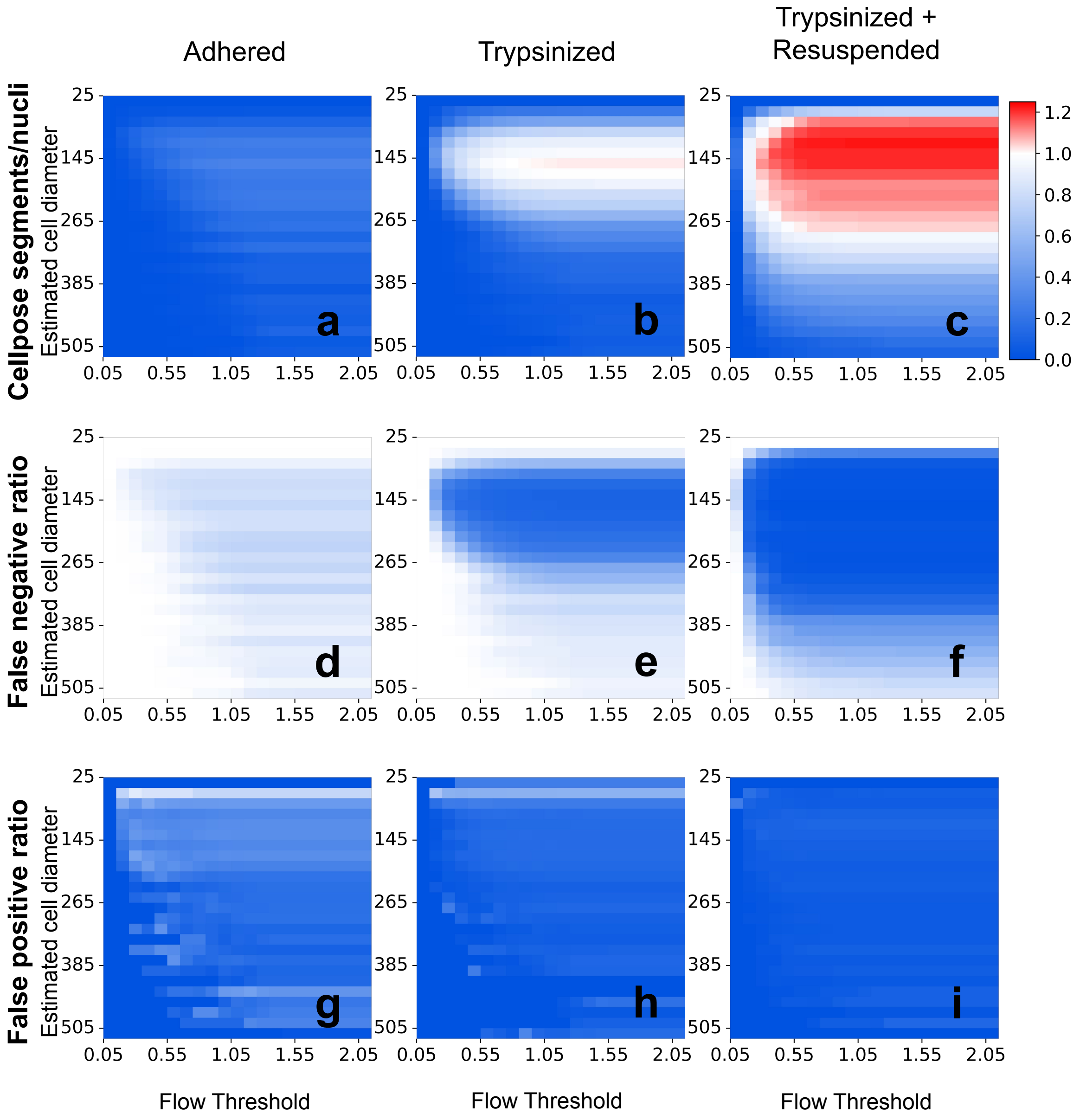

Supplementary figure S1 Cellpose parameter optimization.

a-c. Heat map of Cellpose segments per number of counted nuclei for all combinations of estimated cell diameter and flow threshold assessed for adhered (left), trypsinized (middle), and trypsinized + resuspended (right) condition. **d-f**. Same as **a-c** for false negative ratio. **g-i**. Same as **a-c** for false positive ratio.

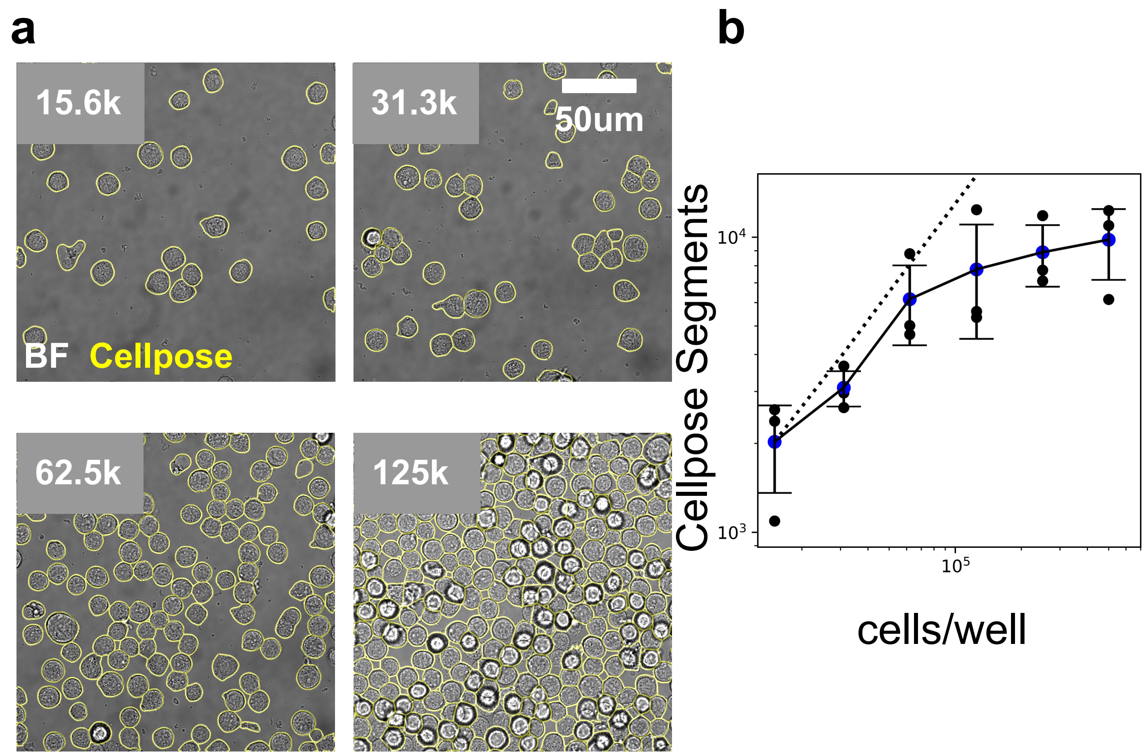

Supplementary figure S2 Cell density optimization for Cellpose segmentation
a. Representative images and Cellpose segment outlines for 15.6k, 31.3k, 62.5k, and 125k cells/well of a 96 well plate. **b**. Cellpose segments obtained from 49 images for varying number of loaded cells. Dotted line is the theoretical trend expected if Cellpose segments and number of loaded cells were perfectly correlated.

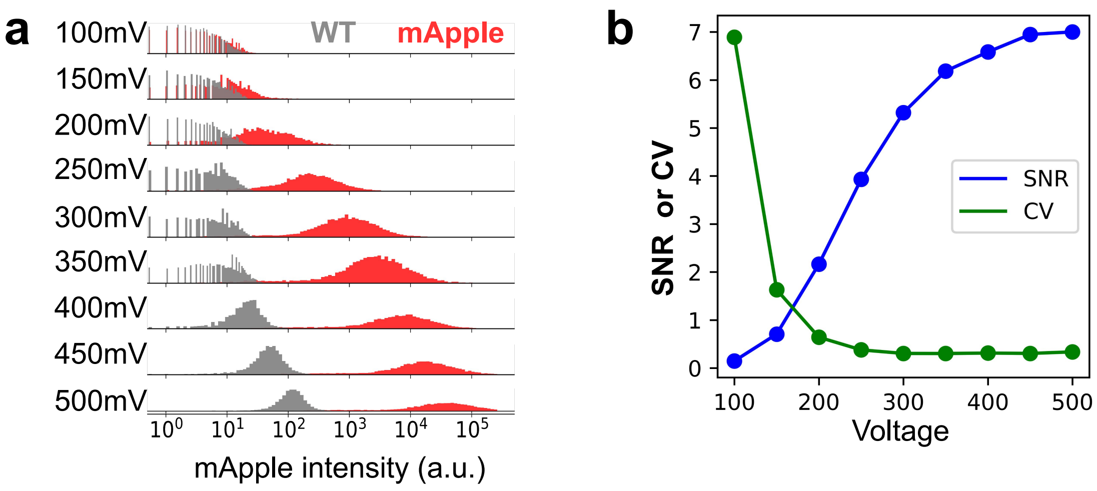

Supplementary figure S3 Flow cytometry optimization
a. Histogram of mApple+ and non fluorescent (WT) cell's fluorescent intensity obtained via Flow for varying PMT voltages. **b**. SNR and Coefficient of variation (CV) for data in **a** obtained via flow cytometry.

Plasmid sequences

AASV1_Pa01attb_BFP_puro_HA
ctagtcctgcaggtttaaacgaattcgccctttgctttctctgaccagcattctctcccctgggcctgtgccgctttctgtctgc
agcttgtggcctgggtcacctctacggctggcccagatccttccctgccgcctccttcaggttccgtcttcctccactccctct
tccccttgctctctgctgtgttgctgcccaaggatgctctttccggagcacttccttctcggcgctgcaccacgtgatgtcctct
gagcggatcctccccgtgtctgggtcctctccgggcatctctcctccctcacccaaccccatgccgtcttcactcgctgggt
tccctttccttctccttctggggcctgtgccatctctcgtttcttaggatggccttctccgacggatgtctcccttgcgtcccgcc
tccccttcttgtaggcctgcatcatcaccgtttttctggacaaccccaaagtaccccgtctccctggctttagccacctctcc
atcctcttgctttctttgcctggacaccccgttctcctgtggattcgggtcacctctcactcctttcatttgggcagctcccctac
ccccccttacctctctagtctgtgctagctcttccagccccctgtcatggcatcttccaggggtccgagagctcagctagtctt
cttcctccaacccgggcccctatgtccacttcaggacagcatgtttgctgcctccagggatcctgtgtccccgagctggg
accaccttatattcccagggccggttaatgtggctctggttctgggtacttttatctgtcccctccaccccacagtggggcaa
gcttctgacctcttctcttcctcccacagggcctcgagagatctggcagcggagagggcagaggaagtcttctaacatg
cggtgacgtggaggagaatcccggccctaggctcgagGAGACCGTGACCTACATGCTCGAAGGG
CGTATGCGCCACGAAATGAGCGAGCTGATTAAGGAGAACATGCACATGAAGCTG
TACATGGAGGGCACCGTGGACAACCATCACTTCAAGTGCACATCCGAGGGCGAA
GGCAAGCCCTACGAGGGCACCCAGACCATGAGAATCAAGGTGGTCGAGGGCGG
CCCTCTCCCCTTCGCCTTCGACATCCTGGCTACTAGCTTCCTCTACGGCAGCAA
GACCTTCATCAACCACACCCAGGGCATCCCCGACTTCTTCAAGCAGTCCTTCCC
TGAGGGCTTCACATGGGAGAGAGTCACCACATACGAAGACGGGGGCGTGCTGA
CCGCTACCCAGGACACCAGCCTCCAGGACGGCTGCCTCATCTACAACGTCAAGA
TCAGAGGGGTGAACTTCACATCCAACGGCCCTGTGATGCAGAAGAAAACACTCG
GCTGGGAGGCCTTCACCGAAACGCTGTACCCCGCTGACGGCGGCCTGGAAGGC
AGAAACGACATGGCCCTGAAGCTCGTGGGCGGGAGCCATCTGATCGCAAACAT
CAAGACCACATATAGATCCAAGAAACCCGCTAAGAACCTCAAGATGCCTGGCGT
CTACTATGTGGACTACAGACTGGAAAGAATCAAGGAGGCCAACAACGAAACCTA
CGTCGAGCAGCACGAGGTGGCAGTGGCCAGATACTGCGACCTCCCTAGCAAAC
TGGGGCACAAGCTTAATCCCAAGAAGAAGAGGAAGGTGgagggcagaggaagtcttctaa
catgcggtgacgtggaggagaatcccggccctaggctcgagatgaccgagtacaagcccacggtgcgcctcgcca
cccgcgacgacgtccccagggccgtacgcaccctcgccgccgcgttcgccgactaccccgccacgcgccacaccg
tcgatccggaccgccacatcgagcgggtcaccgagctgcaagaactcttcctcacgcgcgtcgggctcgacatcggc
aaggtgtgggtcgcggacgacggcgccgcggtggcggtctggaccacgccggagagcgtcgaagcgggggcggt
gttcgccgagatcggcccgcgcatggccgagttgagcggttcccggctggccgcgcagcaacagatggaaggcctc
ctggcgccgcaccggcccaaggagcccgcgtggttcctggccaccgtcggcgtctcgcccgaccaccagggcaag
ggtctgggcagcgccgtcgtgctccccggagtggaggcggccgagcgcgccggggtgcccgccttcctggagacct
ccgcgccccgcaacctccccttctacgagcggctcggcttcaccgtcaccgccgacgtcgaggtgcccgaaggacc
gcgcacctggtgcatgacccgcaagcccggtgcctgaCTCGAGCGACGCCTCGACTGTGCCTTCT
AGTTGCCAGCCATCTGTTGTTTGCCCCTCCCCCGTGCCTTCCTTGACCCTGGAA
GGTGCCACTCCCACTGTCCTTTCCTAATAAAATGAGGAAATTGCATCGCATTGTC
TGAGTAGGTGTCATTCTATTCTGGGGGGTGGGGTGGGGCAGGACAGCAAGGGG
GAGGATTGGGAAGACAATAGCAGGCATGCTGGGGATCCacgactgacaggattggtgaca
gaaaagccccatccttaggcctcctccttcctagtctcctgatattgggtctaacccccacctcctgttaggcagattcctta
tctggtgacacacccccatttcctggagccatctctctccttgccagaacctctaaggtttgcttacgatggagccagaga
ggatcctgggagggagagcttggcagggggtgggagggaagggggggatgcgtgacctgcccggttctcagtggc
caccctgcgctaccctctcccagaacctgagctgctctgacgcggctgtctggtgcgtttcactgatcctggtgctgcagc
ttccttacacttcccaagaggagaagcagtttggaaaaacaaaatcagaataagttggtcctgagttctaactttggctctt
cacctttctagtccccaatttatattgttcctccgtgcgtcagttttacctgtgagataaggccagtagccagccccgtcctgg
cagggctgtggtgaggagggggtgtccgtgtggaaaactccctttgtgagaatggtgcgtcctaggtgttcaccaggt
cgtggccgcctctactccctttctctttctccatccttctttccttaaagagtccccagtgctatctgggacatattcctccgccc
agagcagggtcccgcttccctaaggccctgctctgggcttctgggtttgagtccttggcaagcccaggagaggcgctca

ggcttccctgtccccttcctcgtccaccatctcatgcccctggctctcctgccccttccctacaggggttcctggctctgctct
aagggcgaattcgcggccgctaaattcaattcgccctatagtgagtcgtattacaattcactggccgtcgttttacaacgtc
gtgactgggaaaaccctggcgttacccaacttaatcgccttgcagcacatcccccttcgccagctggcgtaatagcga
agaggcccgcaccgatcgcccttcccaacagttgcgcagcctatacgtacggcagtttaaggtttacacctataaaag
agagagccgttatcgtctgtttgtggatgtacagagtgatattattgacacgccggggcgacggatggtgatccccctgg
ccagtgcacgtctgctgtcagataaagtctcccgtgaactttacccggtggtgcatatcggggatgaaagctggcgcat
gatgaccaccgatatggccagtgtgccggtctccgttatcggggaagaagtggctgatctcagccaccgcgaaaatg
acatcaaaaacgccattaacctgatgttctggggaatataaatgtcaggcatgagattatcaaaaaggatcttcaccta
gatccttttcacgtagaaagccagtccgcagaaacggtgctgaccccggatgaatgtcagctactgggctatctggaca
agggaaaacgcaagcgcaaagagaaagcaggtagcttgcagtgggcttacatggcgatagctagactgggcggttt
tatggacagcaagcgaaccggaattgccagctggggcgccctctggtaaggttgggaagccctgcaaagtaaactg
gatggctttcttgccgccaaggatctgatggcgcaggggatcaagctctgatcaagagacaggatgaggatcgtttcgc
atgattgaacaagatggattgcacgcaggttctccggccgcttgggtggagaggctattcggctatgactgggcacaac
agacaatcggctgctctgatgccgccgtgttccggctgtcagcgcaggggcgcccggttctttttgtcaagaccgacctg
tccggtgccctgaatgaactgcaagacgaggcagcgcggctatcgtggctggccacgacgggcgttccttgcgcagc
tgtgctcgacgttgtcactgaagcgggaagggactggctgctattgggcgaagtgccggggcaggatctcctgtcatct
caccttgctcctgccgagaaagtatccatcatggctgatgcaatgcggcggctgcatacgcttgatccggctacctgccc
attcgaccaccaagcgaaacatcgcatcgagcgagcacgtactcggatggaagccggtcttgtcgatcaggatgatct
ggacgaagagcatcaggggctcgcgccagccgaactgttcgccaggctcaaggcgagcatgcccgacggcgagg
atctcgtcgtgacccatgcatggcgatgcctgcttgccgaatatcatggtggaaaatggccgcttttctggattcatcgact
gtggccggctgggtgtggcggaccgctatcaggacatagcgttggctacccgtgatattgctgaagagcttggcggcg
aatgggctgaccgcttcctcgtgctttacggtatcgccgctcccgattcgcagcgcatcgccttctatcgccttcttgacga
gttcttctgaattattaacgcttacaatttcctgatgcggtattttctccttacgcatctgtgcggtatttcacaccgcatcaggtg
gcacttttcggggaaatgtgcgcggaacccctatttgtttatttttctaaatacattcaaatatgtatccgctcatgagattatc
aaaaaggatcttcacctagatccttttaaattaaaaatgaagttttaaatcaatctaaagtatatatgagtaaacttggtctg
acagttaccaatgcttaatcagtgaggcacctatctcagcgatctgtctatttcgttcatccatagttgcctgactccccgtc
gtgtagataactacgatacgggagggcttaccatctggccccagtgctgcaatgataccgcgagacccacgctcacc
ggctccagatttatcagcaataaaccagccagccggaagggccgagcgcagaagtggtcctgcaactttatccgcct
ccatccagtctattaattgttgccgggaagctagagtaagtagttcgccagttaatagtttgcgcaacgttgttgccattgct
acaggcatcgtggtgtcacgctcgtcgtttggtatggcttcattcagctccggttcccaacgatcaaggcgagttacatgat
cccccatgttgtgcaaaaaagcggttagctccttcggtcctccgatcgttgtcagaagtaagttggccgcagtgttatcact
catggttatggcagcactgcataattctcttactgtcatgccatccgtaagatgcttttctgtgactggtgagtactcaacca
agtcattctgagaatagtgtatgcggcgaccgagttgctcttgcccggcgtcaatacgggataataccgcgccacatag
cagaactttaaaagtgctcatcattggaaaacgttcttcggggcgaaaactctcaaggatcttaccgctgttgagatcca
gttcgatgtaacccactcgtgcacccaactgatcttcagcatcttttactttcaccagcgtttctgggtgagcaaaaacagg
aaggcaaaatgccgcaaaaaagggaataagggcgacacggaaatgttgaatactcatactcttcctttttcaatattatt
gaagcatttatcagggttattgtctcatgaccaaaatcccttaacgtgagttttcgttccactgagcgtcagaccccgtaga
aaagatcaaaggatcttcttgagatcctttttttctgcgcgtaatctgctgcttgcaaacaaaaaaaccaccgctaccagc
ggtggtttgtttgccggatcaagagctaccaactctttttccgaaggtaactggcttcagcagagcgcagataccaaata
ctgttcttctagtgtagccgtagttaggccaccacttcaagaactctgtagcaccgcctacatacctcgctctgctaatcctg
ttaccagtggctgctgccagtggcgataagtcgtgtcttaccgggttggactcaagacgatagttaccggataaggcgc
agcggtcgggctgaacggggggttcgtgcacacagcccagcttggagcgaacgacctacaccgaactgagatacct
acagcgtgagctatgagaaagcgccacgcttcccgaagggagaaaggcggacaggtatccggtaagcggcaggg
tcggaacaggagagcgcacgagggagcttccagggggaaacgcctggtatctttatagtcctgtcgggtttcgccacc
tctgacttgagcgtcgatttttgtgatgctcgtcaggggggcggagcctatggaaaaacgccagcaacgcggcctttta
cggttcctggccttttgctggccttttgctcacatgttctttcctgcgttatcccctgattctgtggataaccgtattaccgcctttg
agtgagctgataccgctcgccgcagccgaacgaccgagcgcagcgagtcagtgagcgaggaagcggaagagcg
cccaatacgcaaaccgcctctccccgcgcgttggccgattcattaatgcagctggcacgacaggtttcccgactggaa
agcgggcagtgagcgcaacgcaattaatgtgagttagctcactcattaggcaccccaggctttacactttatgcttccgg
ctcgtatgttgtgtggaattgtgagcggataacaatttcacacaggaaacagctatgaccatgattacgccaagctcaga
attaaccctcactaaaggga

U6_AASV1_HA_sgRNA
gaagatcctttgatcttttctacggggtctgacgctcagtggaacgaaaactcacgttaagggattttggtcatgagattat
caaaaaggatcttcacctagatccttttaaattaaaaatgaagttttaaatcaatctaaagtatatatgagtaaacttggtct
gacagttaccaatgcttaatcagtgaggcacctatctcagcgatctgtctatttcgttcatccatagttgcctgactccccgt
cgtgtagataactacgatacgggagggcttaccatctggccccagtgctgcaatgataccgcgagatccacgctcacc
ggctccagatttatcagcaataaaccagccagccggaagggccgagcgcagaagtggtcctgcaactttatccgcct
ccatccagtctattaattgttgccgggaagctagagtaagtagttcgccagttaatagtttgcgcaacgttgttgccattgct
acaggcatcgtggtgtcacgctcgtcgtttggtatggcttcattcagctccggttcccaacgatcaaggcgagttacatgat
cccccatgttgtgcaaaaaagcggttagctccttcggtcctccgatcgttgtcagaagtaagttggccgcagtgttatcact
catggttatggcagcactgcataattctcttactgtcatgccatccgtaagatgcttttctgtgactggtgagtactcaacca
agtcattctgagaatagtgtatgcggcgaccgagttgctcttgcccggcgtcaatacgggataataccgcgccacatag
cagaactttaaaagtgctcatcattggaaaacgttcttcggggcgaaaactctcaaggatcttaccgctgttgagatcca
gttcgatgtaacccactcgtgcacccaactgatcttcagcatcttttactttcaccagcgtttctgggtgagcaaaaacagg
aaggcaaaatgccgcaaaaaagggaataagggcgacacggaaatgttgaatactcatactcttcctttttcaatattatt
gaagcatttatcagggttattgtctcatgagcggatacatatttgaatgtatttagaaaaataaacaaataggggttccgc
gcacatttccccgaaaagtgccacctgacgtcgctagctgtacaaaaaagcaggctttaaaggaaccaattcagtcga
ctggatccggtaccaaggtcgggcaggaagagggcctatttcccatgattccttcatatttgcatatacgatacaaggct
gttagagagataattagaattaatttgactgtaaacacaaagatattagtacaaaatacgtgacgtagaaagtaataattt
cttgggtagtttgcagttttaaaattatgttttaaaatggactatcatatgcttaccgtaacttgaaagtatttcgatttcttggcttt
atatatcttgtggaaaggacgaaacaccgcaccccacagtggggccaCTgttttagagctagaaatagcaagttaaa
ataaggctagtccgttatcaacttgaaaaagtggcaccgagtcggtgctttttttaagcttgggccgctcgaggtacctctc
tacatatgacatgtgagcaaaaggccagcaaaaggccaggaaccgtaaaaaggccgcgttgctggcgtttttccata
ggctccgcccccctgacgagcatcacaaaaatcgacgctcaagtcagaggtggcgaaacccgacaggactataaa
gataccaggcgtttccccctggaagctccctcgtgcgctctcctgttccgaccctgccgcttaccggatacctgtccgcctt
tctcccttcgggaagcgtggcgctttctcatagctcacgctgtaggtatctcagttcggtgtaggtcgttcgctccaagctgg
gctgtgtgcacgaaccccccgttcagcccgaccgctgcgccttatccggtaactatcgtcttgagtccaacccggtaag
acacgacttatcgccactggcagcagccactggtaacaggattagcagagcgaggtatgtaggcggtgctacagagt
tcttgaagtggtggcctaactacggctacactagaagaacagtatttggtatctgcgctctgctgaagccagttaccttcg
gaaaaagagttggtagctcttgatccggcaaacaaaccaccgctggtagcggtggtttttttgtttgcaagcagcagatt
acgcgcagaaaaaaaggatctcaa

Pa01-attp.1-mApple-polyA
AAAAGGACAATTACAAACAGGAATCGAATGCAACCGGCGCAGGAACACTGCCAG
CGCATCAACAATATTTTCACCTGAATCAGGATATTCTTCTAATACCTGGAATGCTG
TTTTCCCGGGGATCGCAGTGGTGAGTAACCATGCATCATCAGGAGTACGGATAA
AATGCTTGATGGTCGGAAGAGGCATAAATTCCGTCAGCCAGTTTAGTCTGACCAT
CTCATCTGTAACATCATTGGCAACGCTACCTTTGCCATGTTTCAGAAACAACTCT
GGCGCATCGGGCTTCCCATACAATCGATAGATTGTCGCACCTGATTGCCCGACA
TTATCGCGAGCCCATTTATACCCATATAAATCAGCATCCATGTTGGAATTTAATCG
CGGCCTGGAGCAAGACGTTTCCCGTTGAATATGGCTCATAACACCCCTTGTATTA
CTGTTTATGTAAGCAGACAGTTTTATTGTTCATGATGATATATTTTTATCTTGTGCA
ATGTAACATCAGAGATTTTGAGACACAACGTGGCTTTGTTGAATAAATCGAACTTT
TGCTGAGTTGAAGGATCAGTCATGACCAAAATCCCTTAACGTGAGTTTTCGTTCC
ACTGAGCGTCAGACCCCGTAGAAAAGATCAAAGGATCTTCTTGAGATCCTTTTTT
TCTGCGCGTAATCTGCTGCTTGCAAACAAAAAAACCACCGCTACCAGCGGTGGT
TTGTTTGCCGGATCAAGAGCTACCAACTCTTTTTCCGAAGGTAACTGGCTTCAGC
AGAGCGCAGATACCAAATACTGTTCTTCTAGTGTAGCCGTAGTTAGGCCACCACT
TCAAGAACTCTGTAGCACCGCCTACATACCTCGCTCTGCTAATCCTGTTACCAGT
GGCTGCTGCCAGTGGCGATAAGTCGTGTCTTACCGGGTTGGACTCAAGACGATA
GTTACCGGATAAGGCGCAGCGGTCGGGCTGAACGGGGGGTTCGTGCACACAGC
CCAGCTTGGAGCGAACGACCTACACCGAACTGAGATACCTACAGCGTGAGCTAT
GAGAAAGC

GCCACGCTTCCCGAAGGGAGAAAGGCGGACAGGTATCCGGTAAGCGGCAGGGT
CGGAACAGGAGAGCGCACGAGGGAGCTTCCAGGGGGAAACGCCTGGTATCTTT
ATAGTCCTGTCGGGTTTCGCCACCTCTGACTTGAGCGTCGATTTTTGTGATGCTC
GTCAGGGGGGCGGAGCCTATGGAAAAACGCCAGCAACGCGGCCTTTTTACGGT
TCCTGGCCTTTTGCTGGCCTTTTGCTCACATGTTCTTTCCTGCGTTATCCCCTGA
TTCTGTGGATAACCGTGCGGCCGCCAATATAACTTCGTATAATGTATGCTATACG
AAGTTATCCCTGAATTCGCATCTAGACTGAACTGGCCGATAATTGCAGACGAGGA
GCATCGCCCTTCCCCGGCCCTCAGGTAAGAGGACCAAATACCGTAGCCGTTTCC
AATTTCAGTCCTTTAGCGCCACCTGGTGCTAACTACTCTATCACGCTTTTATCCAA
TAACTACCTTTGTAAATGTAACGCTCTTCGAGAAAGCAGATTCTCATATCCATCTT
GAGTCTTCTTTCTCGCAAGACAACACGAAATAGACACAGTCTCTTCCCTAGCTGT
ACACTGtGCCGTGAGCAAGGGCGAGGAGAATAACATGGCCATCATCAAGGAGTT
CATGCGCTTCAAGGTGCACATGGAGGGCTCCGTGAACGGCCACGAGTTCGAGA
TCGAGGGCGAGGGCGAGGGCCGCCCCTACGAGGCCTTTCAGACCGCTAAGCT
GAAGGTGACCAAGGGTGGCCCCCTGCCCTTCGCCTGGGACATCCTGTCCCCTC
AGTTCATGTACGGCTCCAAGGTCTACATTAAGCACCCAGCCGACATCCCCGACT
ACTTCAAGCTGTCCTTCCCCGAGGGCTTCAGGTGGGAGCGCGTGATGAACTTCG
AGGACGGCGGCATTATTCACGTTAACCAGGACTCCTCCCTGCAGGACGGCGTGT
TCATCTACAAGGTGAAGCTGCGCGGCACCAACTTCCCCTCCGACGGCCCCGTAA
TGCAGAAGAAGACCATGGGCTGGGAGGCCTCCGAGGAGCGGATGTACCCCGAG
GACGGCGCCCTGAAGAGCGAGATCAAGAAGAGGCTGAAGCTGAAGGACGGCG
GCCACTACGCCGCCGAGGTCAAGACCACCTACAAGGCCAAGAAGCCCGTGCAG
CTGCCCGGCGCCTACATCGTCGACATCAAGTTGGACATCGTGTCCCACAACGAG
GACTACACCATCGTGGAACAGTACGAACGCGCCGAGGGCCGCCACTCCACCGG
CGGCATGGACGAGCTGTACAAGTAGCTCGAGCGACGCCTCGACTGTGCCTTCTA
GTTGCCAGCCATCTGTTGTTTGCCCCTCCCCCGTGCCTTCCTTGACCCTGGAAG
GTGCCACTCCCACTGTCCTTTCCTAATAAAATGAGGAAATTGCATCGCATTGTCT
GAGTAGGTGTCATTCTATTCTGGGGGGTGGGGTGGGGCAGGACAGCAAGGGGG
AGGATTGGGAAGACAATAGCAGGCATGCTGGGGATGCGGTGGGCTCTATCCGA
GCGGCCGCGTGTTACAACCAATTAACCAATTCTGATTAGAAAAACTCATCGAGCA
TCAAATGAAACTGCAATTTATTCATATCAGGATTATCAATACCATATTTTTGAAAAA
GCCGTTTCTGTAATGAAGGAGAAAACTCACCGAGGCAGTTCCATAGGATGGCAA
GATCCTGGTATCGGTCTGCGATTCCGACTCGTCCAACATCAATACAACCTATTAA
TTTCCCCTCGTCAAAAATAAGGTTATCAAGTGAGAAATCACCATGAGTGACGACT
GAATCCGGTGAGAATGGCAAAAGCTTATGCATTTCTTTCCAGACTTGTTCAACAG
GCCAGCCATTACGCTCGTCATCAAAATCACTCGCATCAACCAAACCGTTATTCAT
TCGTGATTGCGCCTGAGCGAGGCGAAATACGCGATCGCTGTT

NACTB_pegRNA

gaagatcctttgatcttttctacggggtctgacgctcagtggaacgaaaactcacgttaagggattttggtcatgagattat
caaaaaggatcttcacctagatccttttaaattaaaaatgaagttttaaatcaatctaaagtatatatgagtaaacttggtct
gacagttaccaatgcttaatcagtgaggcacctatctcagcgatctgtctatttcgttcatccatagttgcctgactccccgt
cgtgtagataactacgatacgggagggcttaccatctggccccagtgctgcaatgataccgcgagatccacgctcacc
ggctccagatttatcagcaataaaccagccagccggaagggccgagcgcagaagtggtcctgcaactttatccgcct
ccatccagtctattaattgttgccgggaagctagagtaagtagttcgccagttaatagtttgcgcaacgttgttgccattgct
acaggcatcgtggtgtcacgctcgtcgtttggtatggcttcattcagctccggttcccaacgatcaaggcgagttacatgat
cccccatgttgtgcaaaaaagcggttagctccttcggtcctccgatcgttgtcagaagtaagttggccgcagtgttatcact
catggttatggcagcactgcataattctcttactgtcatgccatccgtaagatgcttttctgtgactggtgagtactcaacca
agtcattctgagaatagtgtatgcggcgaccgagttgctcttgcccggcgtcaatacgggataataccgcgccacatag
cagaactttaaaagtgctcatcattggaaaacgttcttcggggcgaaaactctcaaggatcttaccgctgttgagatcca

gttcgatgtaacccactcgtgcacccaactgatcttcagcatcttttactttcaccagcgtttctgggtgagcaaaaacagg
aaggcaaaatgccgcaaaaaagggaataagggcgacacggaaatgttgaatactcatactcttcctttttcaatattatt
gaagcatttatcagggttattgtctcatgagcggatacatatttgaatgtatttagaaaaataaacaaataggggttccgc
gcacatttccccgaaaagtgccacctgacgtcgctagctgtacaaaaaagcaggctttaaaggaaccaattcagtcga
ctggatccggtaccaaggtcgggcaggaagagggcctatttcccatgattccttcatatttgcatatacgatacaaggct
gttagagagataattagaattaatttgactgtaaacacaaagatattagtacaaaatacgtgacgtagaaagtaataattt
cttgggtagtttgcagttttaaaattatgttttaaaatggactatcatatgcttaccgtaacttgaaagtatttcgatttcttggcttt
atatatcttgtggaaaggacgaaacaccGCTATTCTCGCAGCTCACCAgttttagagctagaaatagcaa
gttaaaataaggctagtccgttatcaacttgaaaaagtggcaccgagtcggtgcGACGAGCGCGGCGATA
TCATCATCCATGGtgccgccCATCATATCGGTAAAGGCCTTTTGCCACTCCTTGAAG
TTGAGCTCGGTCATTGAGCTGCGAGAAtttttttaagcttgggccgctcgaggtacctctctacatatga
catgtgagcaaaaggccagcaaaaggccaggaaccgtaaaaaggccgcgttgctggcgtttttccataggctccgcc
cccctgacgagcatcacaaaaatcgacgctcaagtcagaggtggcgaaacccgacaggactataaagataccagg
cgtttccccctggaagctccctcgtgcgctctcctgttccgaccctgccgcttaccggatacctgtccgcctttctcccttcgg
gaagcgtggcgctttctcatagctcacgctgtaggtatctcagttcggtgtaggtcgttcgctccaagctgggctgtgtgca
cgaaccccccgttcagcccgaccgctgcgccttatccggtaactatcgtcttgagtccaacccggtaagacacgactta
tcgccactggcagcagccactggtaacaggattagcagagcgaggtatgtaggcggtgctacagagttcttgaagtgg
tggcctaactacggctacactagaagaacagtatttggtatctgcgctctgctgaagccagttaccttcggaaaaagagt
tggtagctcttgatccggcaaacaaaccaccgctggtagcggtggtttttttgtttgcaagcagcagattacgcgcagaa
aaaaaggatctcaa

U6_ACTB-N_sgRNA

gaagatcctttgatcttttctacggggtctgacgctcagtggaacgaaaactcacgttaagggattttggtcatgagattat
caaaaaggatcttcacctagatccttttaaattaaaaatgaagttttaaatcaatctaaagtatatatgagtaaacttggtct
gacagttaccaatgcttaatcagtgaggcacctatctcagcgatctgtctatttcgttcatccatagttgcctgactccccgt
cgtgtagataactacgatacgggagggcttaccatctggccccagtgctgcaatgataccgcgagatccacgctcacc
ggctccagatttatcagcaataaaccagccagccggaagggccgagcgcagaagtggtcctgcaactttatccgcct
ccatccagtctattaattgttgccgggaagctagagtaagtagttcgccagttaatagtttgcgcaacgttgttgccattgct
acaggcatcgtggtgtcacgctcgtcgtttggtatggcttcattcagctccggttcccaacgatcaaggcgagttacatgat
cccccatgttgtgcaaaaaagcggttagctccttcggtcctccgatcgttgtcagaagtaagttggccgcagtgttatcact
catggttatggcagcactgcataattctcttactgtcatgccatccgtaagatgcttttctgtgactggtgagtactcaacca
agtcattctgagaatagtgtatgcggcgaccgagttgctcttgcccggcgtcaatacgggataataccgcgccacatag
cagaactttaaaagtgctcatcattggaaaacgttcttcggggcgaaaactctcaaggatcttaccgctgttgagatcca
gttcgatgtaacccactcgtgcacccaactgatcttcagcatcttttactttcaccagcgtttctgggtgagcaaaaacagg
aaggcaaaatgccgcaaaaaagggaataagggcgacacggaaatgttgaatactcatactcttcctttttcaatattatt
gaagcatttatcagggttattgtctcatgagcggatacatatttgaatgtatttagaaaaataaacaaataggggttccgc
gcacatttccccgaaaagtgccacctgacgtcgctagctgtacaaaaaagcaggctttaaaggaaccaattcagtcga
ctggatccggtaccaaggtcgggcaggaagagggcctatttcccatgattccttcatatttgcatatacgatacaaggct
gttagagagataattagaattaatttgactgtaaacacaaagatattagtacaaaatacgtgacgtagaaagtaataattt
cttgggtagtttgcagttttaaaattatgttttaaaatggactatcatatgcttaccgtaacttgaaagtatttcgatttcttggcttt
atatatcttgtggaaaggacgaaacaccGCTATTCTCGCAGCTCACCAgttttagagctagaaatagcaa
gttaaaataaggctagtccgttatcaacttgaaaaagtggcaccgagtcggtgctttttttaagcttgggccgctcgaggt
acctctctacatatgacatgtgagcaaaaggccagcaaaaggccaggaaccgtaaaaaggccgcgttgctggcgttt
ttccataggctccgcccccctgacgagcatcacaaaaatcgacgctcaagtcagaggtggcgaaacccgacaggac
tataaagataccaggcgtttccccctggaagctccctcgtgcgctctcctgttccgaccctgccgcttaccggatacctgtc
cgcctttctcccttcgggaagcgtggcgctttctcatagctcacgctgtaggtatctcagttcggtgtaggtcgttcgctcca
agctgggctgtgtgcacgaaccccccgttcagcccgaccgctgcgccttatccggtaactatcgtcttgagtccaaccc
ggtaagacacgacttatcgccactggcagcagccactggtaacaggattagcagagcgaggtatgtaggcggtgcta
cagagttcttgaagtggtggcctaactacggctacactagaagaacagtatttggtatctgcgctctgctgaagccagtta
ccttcggaaaaagagttggtagctcttgatccggcaaacaaaccaccgctggtagcggtggtttttttgtttgcaagcagc
agattacgcgcagaaaaaaaggatctcaa

pCMV-PEmax-P2A

gacattgattattgactagttattaatagtaatcaattacggggtcattagttcatagcccatatatggagttccgcgttacat
aacttacggtaaatggcccgcctggctgaccgcccaacgacccccgcccattgacgtcaataatgacgtatgttcccat
agtaacgccaatagggactttccattgacgtcaatgggtggagtatttacggtaaactgcccacttggcagtacatcaag
tgtatcatatgccaagtacgccccctattgacgtcaatgacggtaaatggcccgcctggcattatgcccagtacatgacc
ttatgggactttcctacttggcagtacatctacgtattagtcatcgctattaccatggtgatgcggttttggcagtacatcaatg
ggcgtggatagcggtttgactcacggggatttccaagtctccaccccattgacgtcaatgggagtttgttttggcaccaaa
atcaacgggactttccaaaatgtcgtaacaactccgccccattgacgcaaatgggcggtaggcgtgtacggtgggag
gtctatataagcagagctggtttagtgaaccgtcagatccgctagagatccgcggccgctaatacgactcactataggg
agagccgccaccatgaaacggacagccgacggaagcgagttcgagtcaccaaagaagaagcggaaagtcgac
aagaagtacagcatcggcctggacatcggcaccaactctgtgggctgggccgtgatcaccgacgagtacaaggtgc
ccagcaagaaattcaaggtgctgggcaacaccgaccggcacagcatcaagaagaacctgatcggagccctgctgtt
cgacagcggcgaaacagccgaggccacccggctgaagagaaccgccagaagaagatacaccagacggaaga
accggatctgctatctgcaagagatcttcagcaacgagatggccaaggtggacgacagcttcttccacagactggaa
gagtccttcctggtggaagaggataagaagcacgagcggcaccccatcttcggcaacatcgtggacgaggtggcct
accacgagaagtaccccaccatctaccacctgagaaagaaactggtggacagcaccgacaaggccgacctgcgg
ctgatctatctggccctggcccacatgatcaagttccggggccacttcctgatcgagggcgacctgaaccccgacaac
agcgacgtggacaagctgttcatccagctggtgcagacctacaaccagctgttcgaggaaaaccccatcaacgcca
gcggcgtggacgccaaggccatcctgtctgccagactgagcaagagcagaaagctggaaaatctgatcgcccagct
gcccggcgagaagaagaatggcctgttcggaaacctgattgccctgagcctgggcctgacccccaacttcaagagc
aacttcgacctggccgaggatgccaaactgcagctgagcaaggacacctacgacgacgacctggacaacctgctg
gcccagatcggcgaccagtacgccgacctgtttctggccgccaagaacctgtccgacgccatcctgctgagcgacat
cctgagagtgaacaccgagatcaccaaggcccccctgagcgcctctatgatcaagagatacgacgagcaccacca
ggacctgaccctgctgaaagctctcgtgcggcagcagctgcctgagaagtacaaagagattttcttcgaccagagcaa
gaacggctacgccggctacattgacggcggagccagccaggaagagttctacaagttcatcaagcccatcctggaa
aagatggacggcaccgaggaactgctcgtgaagctgaagagagaggacctgctgcggaagcagcggaccttcga
caacggcagcatcccccaccagatccacctgggagagctgcacgccattctgcggcggcaggaagatttttacccatt
cctgaaggacaaccgggaaaagatcgagaagatcctgaccttccgcatcccctactacgtgggccctctggccagg
ggaaacagcagattcgcctggatgaccagaaagagcgaggaaaccatcacccccctggaacttcgaggaagtggtg
gacaagggcgcttccgcccagagcttcatcgagcggatgaccaacttcgataagaacctgcccaacgagaaggtgc
tgcccaagcacagcctgctgtacgagtacttcaccgtgtataacgagctgaccaaagtgaaatacgtgaccgaggga
atgagaaagcccgccttcctgagcggcgagcagaaaaaggccatcgtggacctgctgttcaagaccaaccggaaa
gtgaccgtgaagcagctgaaagaggactacttcaagaaaatcgagtgcttcgactccgtggaaatctccggcgtgga
agatcggttcaacgcctccctgggcacataccacgatctgctgaaaattatcaaggacaaggacttcctggacaatga
ggaaaacgaggacattctggaagatatcgtgctgaccctgacactgtttgaggacagagagatgatcgaggaacgg
ctgaaaacctatgcccacctgttcgacgacaaagtgatgaagcagctgaagcggcggagatacaccggctggggc
aggctgagccggaagctgatcaacggcatccgggacaagcagtccggcaagacaatcctggatttcctgaagtccg
acggcttcgccaacagaaacttcatgcagctgatccacgacgacagcctgacctttaaagaggacatccagaaagc
ccaggtgtccggccagggcgatagcctgcacgagcacattgccaatctggccggcagccccgccattaagaagggc
atcctgcagacagtgaaggtggtggacgagctcgtgaaagtgatgggccggcacaagcccgagaacatcgtgatcg
aaatggccagagagaaccagaccacccagaagggacagaagaacagccgcgagagaatgaagcggatcgaa
gagggcatcaaagagctgggcagccagatcctgaaagaacaccccgtggaaaacacccagctgcagaacgaga
agctgtacctgtactacctgcagaatgggcgggatatgtacgtggaccaggaactggacatcaaccggctgtccgact
acgatgtggacgctatcgtgcctcagagctttctgaaggacgactccatcgacaacaaggtgctgaccagaagcgac
aagaaccggggcaagagcgacaacgtgccctccgaagaggtcgtgaagaagatgaagaactactggcggcagct
gctgaacgccaagctgattacccagagaaagttcgacaatctgaccaaggccgagagaggcggcctgagcgaact
ggataaggccggcttcatcaagagacagctggtggaaacccggcagatcacaaagcacgtggcacagatcctgga
ctcccggatgaacactaagtacgacgagaatgacaagctgatccgggaagtgaaagtgatcaccctgaagtccaag
ctggtgtccgatttccggaaggatttccagttttacaaagtgcgcgagatcaacaactaccaccacgcccacgacgcct
acctgaacgccgtcgtgggaaccgccctgatcaaaaagtaccctaagctggaaagcgagttcgtgtacggcgactac
aaggtgtacgacgtgcggaagatgatcgccaagagcgagcaggaaatcggcaaggctaccgccaagtact

tcttctacagcaacatcatgaactttttcaagaccgagattaccctggccaacggcgagatccggaagcggcctctgat
cgagacaaacggcgaaaccggggagatcgtgtgggataagggccgggattttgccaccgtgcggaaagtgctgag
catgccccaagtgaatatcgtgaaaaagaccgaggtgcagacaggcggcttcagcaaagagtctatcctgcccaag
aggaacagcgataagctgatcgccagaaagaaggactgggaccctaagaagtacggcggcttcgacagccccac
cgtggcctattctgtgctggtggtggccaaagtggaaaagggcaagtccaagaaactgaagagtgtgaaagagctgc
tggggatcaccatcatggaaagaagcagcttcgagaagaatcccatcgactttctggaagccaagggctacaaaga
agtgaaaaaggacctgatcatcaagctgcctaagtactccctgttcgagctggaaaacggccggaagagaatgctgg
cctctgccggcgaactgcagaagggaaacgaactggccctgccctccaaatatgtgaacttcctgtacctggccagcc
actatgagaagctgaagggctcccccgaggataatgagcagaaacagctgtttgtggaacagcacaagcactacct
ggacgagatcatcgagcagatcagcgagttctccaagagagtgatcctggccgacgctaatctggacaaagtgctgt
ccgcctacaacaagcaccgggataagcccatcagagagcaggccgagaatatcatccacctgtttaccctgaccaat
ctgggagcccctgccgccttcaagtactttgacaccaccatcgaccggaagaggtacaccagcaccaaagaggtgct
ggacgccaccctgatccaccagagcatcaccggcctgtacgagacacggatcgacctgtctcagctgggaggtgact
ccggcggaagctctggtggcagcaagcggaccgccgacggctctgaattcgagagccctaagaagaaaagaaag
gtgagcggaggctctagcggcggaagcaccctgaacattgaagacgagtatagactgcatgaaacaagcaagga
acccgacgtgtccctgggctccacctggctgtccgactttccccaggcctgggccgagacaggaggaatgggcctgg
ccgtgcggcaggcacccctgatcatccctctgaaggccacctctacacccgtgagcatcaagcagtaccctatgtctca
ggaggccagactgggcatcaagcctcacatccagaggctgctggaccagggcatcctggtgccatgccagagcccc
tggaacacaccactgctgcccgtgaagaagccaggcaccaatgactatagacccgtgcaggatctgagagaggtg
aacaagagggtggaggatatccaccccaccgtgcccaacccttacaatctgctgtccggcctgcccccttctcaccagt
ggtatacagtgctggacctgaaggatgccttcttttgtctgagactgcaccctaccagccagccactgttcgcctttgagtg
gagggaccctgagatgggcatctctggccagctgacctggacacgcctgcctcagggcttcaagaatagcccaaca
ctgtttaacgaggccctgcaccgcgacctggcagatttccggatccagcacccagatctgatcctgctgcagtacgtgg
acgatctgctgctggccgccaccagcgagctggattgccagcagggaacacgcgccctgctgcagaccctgggaaa
cctgggatatagggcatccgccaagaaggcccagatctgtcagaagcaggtgaagtacctgggctatctgctgaagg
agggccagagatggctgacagaggccaggaaggagacagtgatgggccagccaacacccaagaccccaagac
agctgagggagttcctgggcaaagcaggattttgcaggctgttcatcccaggattcgcagagatggcagcacctctgta
cccactgaccaagccgggcaccctgtttaattggggccctgaccagcagaaggcctatcaggagatcaagcaggcc
ctgctgacagcaccagccctgggcctgccagacctgaccaagcctttcgagctgtttgtggatgagaagcagggctac
gccaagggcgtgctgacccagaagctgggaccatggagacggcccgtggcctatctgtccaagaagctggaccca
gtggcagcaggatggccaccatgcctgaggatggtggcagcaatcgccgtgctgacaaaggatgccggcaagctg
accatgggacagccactggtcatcctggcaccacacgcagtggaggccctggtgaagcagcctccagatcgctggct
gtctaacgcccggatgacacactaccaggccctgctgctggacaccgatcgcgtgcagtttggccctgtggtggccctg
aatccagccaccctgctgcctctgccagaggagggcctgcagcacaactgtctggacatcctggcagaggcacacg
gaacaaggccagacctgaccgatcagcccctgcctgacgccgatcacacatggtataccgatggaagctccctgctg
caggagggccagaggaaggcaggagcagcagtgaccacagagacagaagtgatctgggccaaggccctgcca
gcaggcacatccgcccagcgggccgagctgatcgccctgacccaggccctgaagatggccgagggcaagaagct
gaacgtgtacacagactccagatatgccttcgccaccgcacacatccacggagagatctacaggcgccggggctgg
ctgacctctgagggcaaggagatcaagaacaaggatgagatcctggccctgctgaaggccctgtttctgcccaagcg
gctgagcatcatccactgtcctggacaccagaagggacactccgccgaggcaaggggcaatcggatggccgacca
ggccgccagaaaggctgctattactgaaactcccgacacttccactctgctgattgaaaactcctcccttctggcggct
caaaaagaaccgccgacggcagcgaattcgagtctcccaagaagaagaggaaagtcggctctggccctgccgcta
agagagtgaagctggacggatccggcgcaacaaacttctctctgctgaaacaagccggagatgtcgaagagaatcc
tggaccgccgcatcatcaccatcaccattgagtttaaacccgctgatcagcctcgactgtgccttctagttgccagccatc
tgttgtttgcccctcccccgtgccttccttgaccctggaaggtgccactcccactgtcctttcctaataaaatgagaaattg
catcgcattgtctgagtaggtgtcattctattctggggggtggggtggggcaggacagcaagggggaggattgggaag
acaatagcaggcatgctggggatgcggtgggctctatggcttctgaggcggaaagaaccagctggggctcgataccg
tcgacctctagctagagcttggcgtaatcatggtcatagctgtttcctgtgtgaaattgttatccgctcacaattccacacaa
catacgagccggaagcataaagtgtaaagcctagggtgcctaatgagtgagctaactcacattaattgcgttgcgctca
ctgcccgctttccagtcgggaaacctgtcgtgccagctgcattaatgaatcggccaacgcgcggggagaggcggtttg
cgtattgggcgctcttccgcttcctcgctcactgactcgctgcgctcggtcgttcggctgcggcgagcggtatcag

ctcactcaaaggcggtaatacggttatccacagaatcaggggataacgcaggaaagaacatgtgagcaaaaggcc
agcaaaaggccaggaaccgtaaaaaggccgcgttgctggcgtttttccataggctccgcccccctgacgagcatcac
aaaaatcgacgctcaagtcagaggtggcgaaacccgacaggactataaagataccaggcgtttccccctggaagct
ccctcgtgcgctctcctgttccgaccctgccgcttaccggatacctgtccgcctttctcccttcgggaagcgtggcgctttctc
atagctcacgctgtaggtatctcagttcggtgtaggtcgttcgctccaagctgggctgtgtgcacgaaccccccgttcagc
ccgaccgctgcgccttatccggtaactatcgtcttgagtccaacccggtaagacacgacttatcgccactggcagcagc
cactggtaacaggattagcagagcgaggtatgtaggcggtgctacagagttcttgaagtggtggcctaactacggcta
cactagaagaacagtatttggtatctgcgctctgctgaagccagttaccttcggaaaaagagttggtagctcttgatccgg
caaacaaaccaccgctggtagcggtggtttttttgtttgcaagcagcagattacgcgcagaaaaaaaggatctcaaga
agatcctttgatcttttctacggggtctgacactcagtggaacgaaaactcacgttaagggattttggtcatgagattatca
aaaaggatcttcacctagatccttttaaattaaaaatgaagttttaaatcaatctaaagtatatatgagtaaacttggtctga
cagttaccaatgcttaatcagtgaggcacctatctcagcgatctgtctatttcgttcatccatagttgcctgactccccgtcgt
gtagataactacgatacgggagggcttaccatctggccccagtgctgcaatgataccgcgagacccacgctcaccgg
ctccagatttatcagcaataaaccagccagccggaagggccgagcgcagaagtggtcctgcaactttatccgcctcc
atccagtctattaattgttgccgggaagctagagtaagtagttcgccagttaatagtttgcgcaacgttgttgccattgctac
aggcatcgtggtgtcacgctcgtcgtttggtatggcttcattcagctccggttcccaacgatcaaggcgagttacatgatcc
cccatgttgtgcaaaaaagcggttagctccttcggtcctccgatcgttgtcagaagtaagttggccgcagtgttatcactca
tggttatggcagcactgcataattctcttactgtcatgccatccgtaagatgcttttctgtgactggtgagtactcaaccaagt
cattctgagaatagtgtatgcggcgaccgagttgctcttgcccggcgtcaatacgggataataccgcgccacatagcag
aactttaaaagtgctcatcattggaaaacgttcttcggggcgaaaactctcaaggatcttaccgctgttgagatccagttc
gatgtaacccactcgtgcacccaactgatcttcagcatcttttactttcaccagcgtttctgggtgagcaaaaacaggaag
gcaaaatgccgcaaaaaagggaataagggcgacacggaaatgttgaatactcatactcttcctttttcaatattattgaa
gcatttatcagggttattgtctcatgagcggatacatatttgaatgtatttagaaaaataaacaaataggggttccgcgcac
atttccccgaaaagtgccacctgacgtcgacggatcgggagatcgatctcccgatcccctagggtcgactctcagtaca
atctgctctgatgccgcatagttaagccagtatctgctccctgcttgtgtgttggaggtcgctgagtagtgcgcgagcaaa
atttaagctacaacaaggcaaggcttgaccgacaattgcatgaagaatctgcttagggttaggcgttttgcgctgcttcgc
gatgtacgggccagatatacgcgtt
